# Fabrication and Evaluation of Ceramic-Based Hollow Fiber Membrane Modules for Hemodialysis Applications

**DOI:** 10.3390/membranes15090251

**Published:** 2025-08-26

**Authors:** Jae Yeon Hwang, Sung Woo Han, Seung Hee Huh, So Hee Park, Sang Min Park, Jung Hoon Park

**Affiliations:** Department of Chemical and Biochemical Engineering, Dongguk University, Pildong-ro 1-gil, Jung-gu, Seoul 04620, Republic of Korea; fzytm@naver.com (J.Y.H.); lunadial486@naver.com (S.W.H.); seunghee437@naver.com (S.H.H.); mnk916@naver.com (S.H.P.); 1115pk@naver.com (S.M.P.)

**Keywords:** ceramic hollow fiber, hemodialysis membrane, sintering temperature, uremic toxin removal, biocompatibility, protein adsorption

## Abstract

The application of ceramic membranes in hemodialysis modules remains underexplored, as prior investigations have primarily concentrated on flat-sheet samples or small-scale assessments. This study advances the field by fabricating Al_2_O_3_ hollow fiber membranes, integrating them into a lab-scale module, and systematically evaluating the influence of sintering temperature on their structural characteristics, hemocompatibility, and dialysis performance. Al_2_O_3_ hollow fiber membranes were prepared using a phase inversion method and then sintered at three different temperatures. All membranes exhibited superior protein adsorption behavior compared to conventional polymer-based membranes, which indicates higher biocompatibility. Furthermore, the amount of adsorbed protein decreased with increasing sintering temperature. This suggests that the amount of protein adsorption can be controlled by adjusting the heat treatment conditions. The lab-scale hemodialyzer integrated with a membrane sintered at 1200 °C achieved the fastest urea removal rate of approximately 90% in 2 h and reached a *Kt*/*V* value of 1.1 after 60 min, which is comparable to the performance of commercial polymer-based hemodialyzers.

## 1. Introduction

Membrane technologies enabling precise separation and control of substances in both in vivo and in vitro environments occupy a critical position in the medical field. In particular, medical membranes play essential roles in a wide range of applications, including blood purification systems [1], extracorporeal membrane oxygenation (ECMO) [2], drug delivery systems [3] and tissue engineering [4], thereby contributing significantly to improved patient survival rates and quality of life. Among these technologies, hemodialysis membranes are indispensable therapeutic tools for patients suffering from chronic renal failure. Their clinical demand and importance have become increasingly prominent, especially during the COVID-19 pandemic, which has seen a surge in cases of acute kidney injury (AKI) [5]. Hemodialysis is an extracorporeal purification technique that partially substitutes for renal function by effectively removing metabolic waste products, excess fluids, and correcting electrolyte imbalances that are otherwise not excreted naturally from the body.

The development of hemodialysis membranes began in the 1940s with Kolff’s invention of the first commercial hemodialyzer, which employed cellulose tubing as a semipermeable membrane [6,7,8,9,10]. Although cellulose allowed selective diffusion of small solutes, it triggered immune activation that caused side effects such as thrombocytopenia and leukopenia [11,12,13], and its rigid crystal structure limited microstructural control and functional modification [14]. To overcome these drawbacks, synthetic polymer membranes such as polysulfone (PSF) and polyethersulfone (PES) were introduced in the 1970s, and with their superior stability and tunable properties, they have been established as the standard in hemodialysis since the 1980s [15,16,17,18,19,20,21,22]. Nevertheless, those synthetic polymer-based membranes had another challenge related to biocompatibility. Since most synthetic polymer materials, such as PSF and PES, are basically hydrophobic, they can induce nonspecific protein adsorption, platelet aggregation, and thrombus production when in contact with blood [23,24,25,26]. In particular, when plasma proteins are adsorbed on the surface of the membrane, they may be recognized as foreign substances in the body through structural changes, which may cause an immune response such as inflammation, fever, or allergic reaction. In order to solve this problem, various surface modification methods (hydrophilic polymer, plasma treatment, and adhesive polymer coatings such as PEG and PVP) are being applied. However, residual chemicals from these treatments may induce unintended toxic or immune responses [27,28,29,30,31]. Given these concerns, we investigated ceramic-based membranes as a potential alternative to conventional polymer-based membranes in the field of hemodialysis. Ceramic membranes are structurally stable under high temperature, high pressure, extreme pH conditions, superior chemical stability, thermal resistance, wear resistance, and mechanical strength. These properties are suitable for applications in fields such as CO_2_ capture, industrial wastewater treatment, and hydrogen purification, which require long-term and repetitive performance [32,33,34]. Among many ceramic materials, clumina (Al_2_O_3_), zirconia (ZrO_2_), and titania (TiO_2_) are known to provide excellent biocompatibility and chemical stability and are widely used in biomedical and medical fields, bone transplantation, dental implants and cardiovascular devices [35,36,37]. In addition, the natural hydrophilicity of ceramic surfaces eliminates the need for further surface modification. Their protein adsorption is also much lower than that of polymeric membranes, usually around 0.3 to 0.6 µg/cm^2^ compared with 3 to 8 µg/cm^2^. This lower level of adsorption reduces nonspecific interactions with platelets, and previous studies on ceramic coatings have shown that it can directly suppress platelet adhesion and aggregation. As a result, the risk of thrombosis is reduced, leading to improved hemocompatibility in hemodialysis [38,39,40].

In this context, earlier studies have highlighted the promise of ceramic membranes for hemodialysis. Huang et al. demonstrated that alumina membranes with tailored pore structures enhanced the removal of middle-molecule toxins while limiting albumin loss, and further showed their potential in tubular designs [41,42,43]. Likewise, Roshni et al. found that a low-cost ceramic membrane derived from natural clay exhibited better biocompatibility than commercial polymeric membranes [39,44]. These studies suggest that ceramic membranes can achieve both efficient toxin clearance and good blood compatibility, providing the foundation for the present work. Although ceramic membranes show favorable material properties, as reported by Bender et al. and Czermak et al., there are still technological challenges to overcome. In particular, questions remain about their ability to deliver consistent performance under clinically relevant flow conditions and whether it is possible to scale them into hollow fiber forms, both of which are essential for real-world use in hemodialysis systems [45,46].

The aim of this study is to fabricate hollow fiber type Al_2_O_3_ membranes, which provide a larger surface area within the identical volume compared to flat-sheet and tubular forms, and to assemble a compact lab-scale hemodialysis module to evaluate the applicability. Without additional surface treatment, a protein adsorption test of Al_2_O_3_ hollow fiber membranes was conducted to evaluate biocompatibility. The fabricated lab-scale ceramic hemodialyzer was evaluated for uremic toxin removal, and the performance was quantified using the *Kt*/*V* value, a standard indicator for assessing the adequacy of hemodialysis. This allowed for a prediction of the module’s performance under clinical conditions in comparison to commercial products. To support these findings, various analytical techniques such as SEM, AFM, contact angle measurement, water flux, BCA Assay, UTM, and TOC analysis were conducted.

## 2. Materials and Methods

### 2.1. Materials

Al_2_O_3_ powder (99.9%) was obtained from Kcaracell Co., Ltd. (Daejeon, Republic of Korea) with a mean particle size of 0.5 µm. N-methyl-2-pyrrolidone (NMP, Samchun Pure Chemical Co., Ltd., Seoul, Republic of Korea, 99.5%), Polyethersulfone (PES, Ultrason^®^ E6020, BASF, Ludwigshafen, Germany), and Polyvinylpyrrolidone (PVP, Sigma Aldrich, St. Louis, MA, USA) were used as a solvent, binder, and dispersant of the Al_2_O_3_ hollow fiber solution, respectively. Deionized water was used as both an internal and external coagulant. Bovine Serum Albumin (BSA, Sigma Aldrich, St. Louis, MA, USA), Dodecyl sulfate sodium salt (SDS, Samchun Pure Chemical Co., Ltd., Seoul, Republic of Korea, 85%), Pierce™ BCA Protein Assay Reagents (Thermo Scientific, Waltham, MA, USA) were used for protein adsorption and desorption experiments for membranes. Urea (Samchun Pure Chemical Co., Ltd., Seoul, Republic of Korea, 99.0%) and Creatinine (Sigma Aldrich, St. Louis, MA, USA) were used as uremic toxins in the blood model solution. Considering the normal range of each uremic toxin in the body (Urea: 0.06~0.2 mg/mL, Creatinine: 0.005~0.012 mg/mL), Urea and Creatinine were mixed with distilled water at concentrations of 2 mg/mL and 0.15 mg/mL, respectively.

### 2.2. Preparation of Al_2_O_3_ Hollow Fiber Membranes and Lab-Scale Hemodialyzer

**Figure 1:**
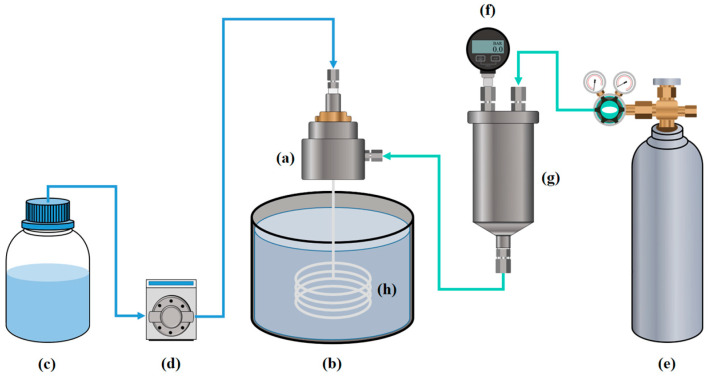
Schematic of the preparation of ceramic hollow fiber membranes: (a) custom made nozzle, (b) external coagulant, (c) internal coagulant, (d) gear pump, (e) air gas, (f) pressure gauge, (g) dope solution tank, (h) green body of the hollow fiber membrane.

The fabrication of ceramic hollow fiber membranes was performed using a phase inversion spinning process and a high-temperature sintering process. A polymer solution including 33.5 wt% of NMP and 6 wt% of PES was uniformly mixed for 24 h at 150 rpm at room temperature. The solution undergoes an additional 24 h of stirring at 300 rpm after adding 60 wt% of Al_2_O_3_ powder with 0.5 wt% of PVP, with the increased stirring speed applied to ensure proper dispersion of the Al_2_O_3_ particles. The completed white mixture was degassed with a vacuum pump (IDP3, Varian, Palo Alto, CA, USA) for an hour. The suspension was extruded by an iron nozzle under a pressure of 3 bars, with a 10 cm air gap maintained between the nozzle tip and the surface of the external coagulant. The internal coagulant flow rate was 20 mL/min, and the spinning of the hollow fiber membranes was carried out at room temperature. The extruded ceramic hollow fiber membranes were immersed in an external coagulant, during which the hollow fiber transitions from a liquid to a solid state. This phase transition process was completed after approximately 1 day. Finally, the hollow fiber membranes were dried at 100 °C for 1 day before being sintered at the various temperatures (1200–1400 °C) for 4 h. Figure 1 shows the schematic of the preparation of ceramic hollow fiber membranes. Heat treatment conditions and a digital photograph of the actual equipment are shown in the Supplementary Materials (Figure S1).

**Figure 2:**
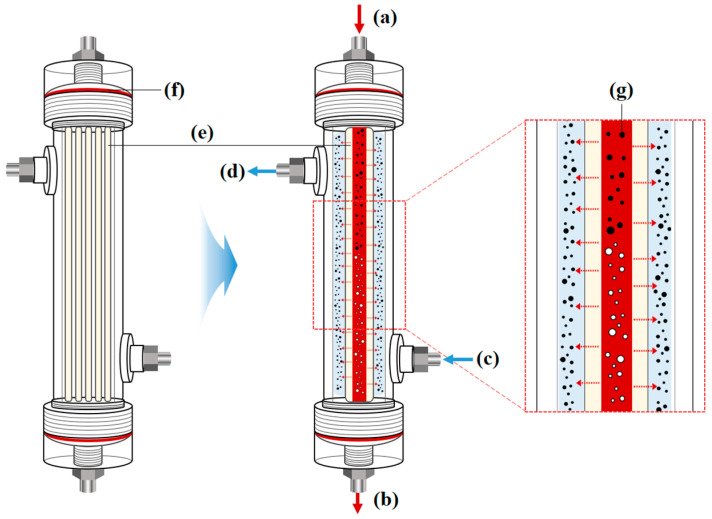
A 2D schematic diagram of the lab-scale hemodialyzer configuration and the principle of uremic toxin removal via diffusion through Al_2_O_3_ hollow fiber membranes: (a) inlet of blood (b) outlet of blood, (c) inlet of dialysate, (d) outlet of dialysate, (e) Al_2_O_3_ hollow fiber membranes, (f) sealed with O-ring, (g) uremic toxins.

The fabricated Al_2_O_3_ hollow fiber membranes were assembled into an acrylic module to produce a lab-scale hemodialyzer. The acrylic module features small holes at each end, allowing for the mounting of hollow fibers. The module was designed to allow dialysate to flow through the shell side and blood through the lumen side of the hollow fiber. The effective membrane surface area of the lab-scale hemodialyzer used in this study is approximately 0.13 m^2^, which corresponds to about one-tenth of the surface area of a commercially available polymer-based hemodialyzer. Figure 2 presents a 2D schematic image of the lab-scale hemodialyzer and an illustration of the hemodialysis process.

### 2.3. Characterization of Al_2_O_3_ Hollow Fiber Membranes and Lab-Scale Hemodialyzer

**Figure 3:**
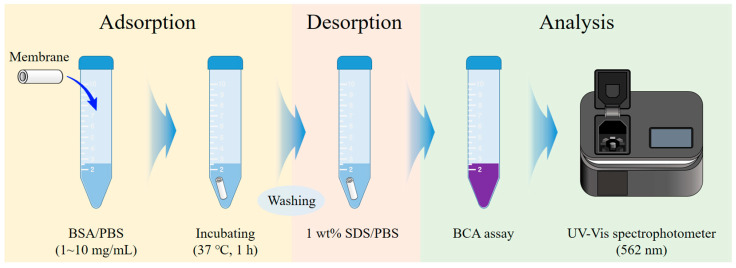
Schematic of the BSA adsorption/desorption process using the BCA assay.

A field emission scanning electron microscopy (FE-SEM, CLARA LMH, TESCAN, Brno, Czech Republic) analysis was performed to observe the surface and cross-sectional structures of the fabricated Al_2_O_3_ hollow fiber membranes. The contact angle was measured using a portable contact angle analyzer (Phoenix-I) to evaluate the surface hydrophilicity depending on the sintering temperature. The volume of the water droplet used for testing the samples was 1 μL. The mechanical strength was measured by a 3-point bending test using a universal testing machine (UTM, K-UTM, Kyung-jin Hitech, Goyang-si, Republic of Korea) to evaluate the mechanical properties of the membranes. The 3-point bending test was performed at a constant strain rate of 10 mm/min, and data were collected until the maximum tensile load was reached. At least 5 measurements were conducted for each sample, and the average flexural strength was calculated. An atomic force microscopy (AFM, NanoMan, Multimode8, Bruker, Billerica, MA, USA) analysis was conducted to evaluate the surface topography and nanoscale roughness of the fabricated membranes. The AFM roughness analysis was carried out using a scan size of 10 × 10 µm^2^ in tapping mode with an operating frequency of approximately 300 kHz, employing an RTESP-300 probe (Sb-doped Si). Surface roughness parameter Ra (average roughness) was extracted from the scanned images to quantitatively assess the nanoscale surface morphology. Through this, the effect on the surface structure and roughness characteristics according to the sintering temperature of the membrane at different temperatures was confirmed. In addition, the biocompatibility of bovine serum albumin (BSA) in Al_2_O_3_ hollow fiber membranes was evaluated through adsorption experiments. The protein adsorption test for the Al_2_O_3_ hollow fiber membranes was conducted as follows. First, the membrane is immersed in a BSA solution based on phosphate-buffered saline (PBS) at a concentration of 1 to 10 mg/mL. Subsequently, the samples were shaken at 200 rpm for 60 min in an incubator set at 37 °C. The membrane was then washed with PBS and then again immersed in 5 mL of PBS containing 0.5 mL of 10% SDS solution to desorb the protein. To quantify the amount of desorbed protein, the Bicinchoninic Acid (BCA) Assay was employed. The BCA Assay is a colorimetric method based on the Biuret reaction, in which peptide bonds (-CONH-) reduce Cu^2+^ ions to Cu^+^ under alkaline conditions. The Cu^+^ ions form a purple-colored complex with two molecules of the BCA reagent. This complex shows strong absorbance at 562 nm, which increases in proportion to the protein concentration. The desorbed protein solution was mixed with the BCA reagent at a ratio of 1:8. A calibration curve was plotted using BSA standard solution samples ranging from 0 to 1.0 mg/mL, prepared in 0.2 mg/mL increments. To account for the effect of SDS on protein quantification, an identical volume of 10% SDS solution was added to all standard samples. By comparing the absorbance with the standard curve of the samples, the amount of BSA adsorbed on the surface of Al_2_O_3_ hollow fiber membrane and subsequently desorbed with SDS could be quantitatively determined. Figure 3 shows the experimental procedure of the BSA adsorption test, and the BSA calibration data is shown in the Supplementary Materials (Figure S2).

**Figure 4:**
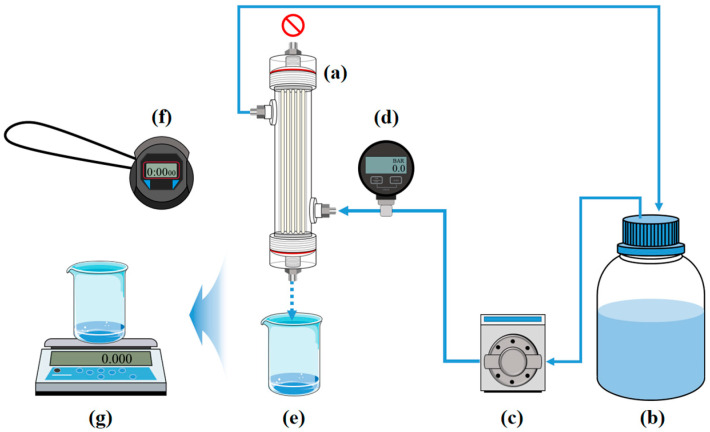
Schematic of the water permeability test system for the lab-scale hemodialyzer: (a) lab-scale hemodialyzer, (b) water tank, (c) gear pump, (d) pressure gauge, (e) permeate, (f) stopwatch, (g) digital scale.

The water permeability of the lab-scale hemodialyzer was evaluated using deionized water. The test was conducted by applying a constant pressure and measuring the weight of the permeated water, and then converting the weight into volume. The membrane module permeability evaluation system utilized the WT3000-1FA liquid pump from LongerPump (China) to circulate distilled water through the dialysate compartment of the lab-scale hemodialyzer. A DPGW-09 pressure gauge from Dwyer (USA) was installed at the inlet of the distilled water supply to monitor and adjust the pressure according to the pump flow rate. The blood compartment was sealed at the top, while a beaker was placed at the bottom to collect the permeated water. The distilled water flowing through the dialysate compartment permeated the hollow fiber membrane surface due to the applied pressure and exited through the bottom of the blood compartment. The applied pressure conditions were set at 0.5, 1.0, 1.5, and 2.0 bar. The overall setup of the water permeability test system for the lab-scale hemodialyzer is shown in Figure 4.

### 2.4. Hemodialysis Experiment of Lab-Scale Hemodialyzer

**Figure 5:**
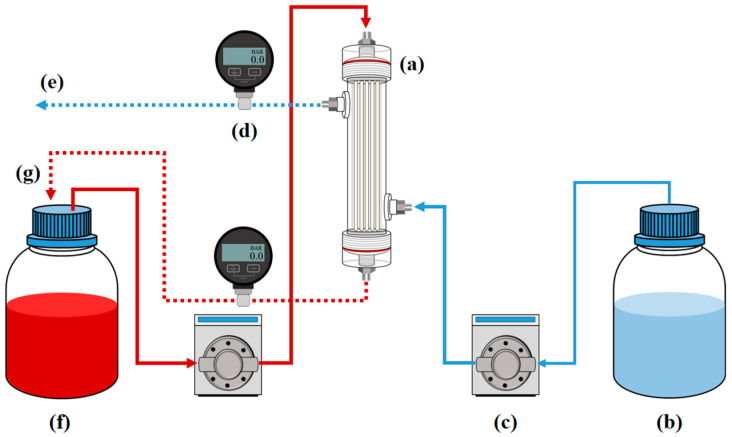
Schematic of the lab-scale hemodialysis experimental system: (a) lab-scale hemodialyzer, (b) dialysate model solution tank, (c) gear pump, (d) pressure gauge, (e) discharge, (f) blood model solution tank, (g) sampling port.

The performance of the lab-scale hemodialyzer was evaluated by circulating a blood model solution through the lumen side of the hollow fiber membranes, while dialysate was flowed counter-currently along the shell side. Solute removal efficiency was quantified by measuring the concentration change of target substances in the blood model and dialysate after dialysis. Based on this fundamental principle, this study conducted hemodialysis experiments using a lab-scale hemodialyzer module including ceramic hollow fiber membranes. As shown in Figure 5, the hemodialysis experimental system was designed to include two liquid pumps. These two pumps were configured to supply liquid separately to the blood compartment and the dialysate compartment. For the blood compartment, the ISM901B pump (ISMATEC, Glattbrugg, Switzerland) was used to supply a blood model solution at a flow rate of approximately 40 mL/min. The blood model solution contains either 3 mg/mL of urea or 0.1 mg/mL of creatinine, which were tested separately to allow quantitative analysis using the TOC method. For the dialysate compartment, a WT3000-1FA pump from LongerPump was used to supply a dialysate model solution at a flow rate of approximately 85 mL/min, and fresh distilled water was continuously supplied as the dialysate model solution. The blood model solution circulated by the lumen side of the hollow fiber membranes, while the dialysate model solution flowed through the shell side of the membranes and was continuously replaced with fresh dialysate. To monitor and control pressure levels, DPGW-09 pressure gauges from Dwyer were installed at the inlets of both compartments. The removal of the uremic toxin should take place by diffusion, not by permeation by pressure. Therefore, the pressures in both compartments remained the same, since an increase in pressure in one compartment could result in ultrafiltration, through which fluid passed through the hollow fiber membrane. The tubing used in this experiment was medical-grade blood tubing from Mecobi Co., Ltd.(Wonju-si, Republic of Korea), connected to the pumps with 1/4-inch fittings. The removal efficiency of uremic toxins by the lab-scale hemodialyzer was evaluated by collecting blood model solution samples at different time intervals. An initial sample was taken before the start of the experiment, followed by additional samples at 1, 2 and 3 h after the start of hemodialysis. The concentration of uremic toxins in these samples was analyzed using a total organic carbon (TOC, Shimadzu, Kyoto, Japan) analyzer.

## 3. Results and Discussion

### 3.1. Structure and Surface Characteristics of Al_2_O_3_ Hollow Fiber Membranes

**Figure 6:**
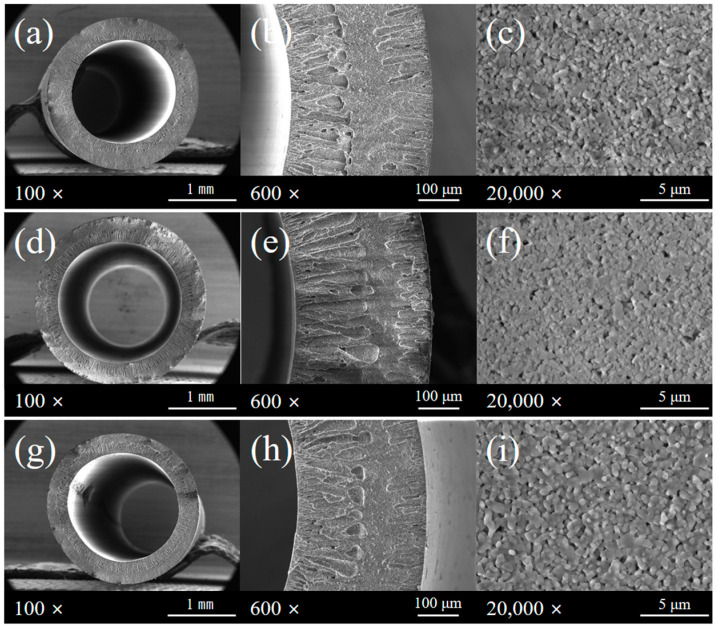
SEM images of Al_2_O_3_ hollow fiber membranes sintered at different temperatures: (**a**–**c**) 1200 °C, (**d**–**f**) 1300 °C, (**g**–**i**) 1400 °C. (**a**,**d**,**g**) Cross-sectional images at 100×, (**b**,**e**,**h**) Higher magnification images at 600×, (**c**,**f**,**i**) Surface microstructures observed at 20,000×.

Scanning electron microscopy (SEM) was performed to analyze the structural characteristics of the Al_2_O_3_ hollow fiber membranes. Figure 6 shows the cross-sectional morphology (Figure 6a,d,g), the magnified inner structure (Figure 6b,e,h), and the surface microstructure (Figure 6c,f,i) of each membrane. The sample sintered at 1200 °C (Figure 6a–c) exhibits a typical asymmetric hollow fiber structure, with numerous finger-like voids. In the high-magnification surface image (Figure 6c), nanoscale pores are uniformly distributed, and an open-pore structure is clearly observed. Such morphology is advantageous for high water permeability and efficient mass transfer performance. The sample sintered at 1300 °C (Figure 6d–f) shows a denser overall structure. In the cross-sectional images (Figure 6d,e), the shell layer appears more compact, and the finger-like voids are fewer and shorter. These changes indicate partial pore shrinkage and closure due to enhanced sintering of alumina particles. The high-resolution surface image (Figure 6f) shows indistinct grain boundaries and a reduced number of visible pores. This indicates the formation of a denser surface. The sample sintered at 1400 °C (Figure 6g–i) exhibits the most compact structure, with a noticeable reduction in membrane thickness. In the cross-sectional images (Figure 6g,h), the finger-like voids on the lumen side became less distinct. In addition, it was confirmed that the sponge structure present between the lumen side and the shell side of the hollow fiber membrane became thinner as the temperature increased. This suggests that the particles agglomerated during the high-temperature sintering process to close the pores and reduce the structure. This can improve durability, mechanical strength and pressure resistance, but it is highly likely to significantly reduce the overall permeability. Overall, increasing the sintering temperature leads to enhanced porosity at 1200 °C, while at 1400 °C the structure becomes denser with significantly lower porosity. The high-resolution surface image (Figure 6i) cannot be clearly different from the surface sintered at the previous two temperatures, but this will be revealed through other analyses performed later.

Water contact angle measurements were performed to evaluate the hydrophilicity of the surface of the Al_2_O_3_ hollow fiber membrane. Figure S3a is the result of measuring the contact angle after placing a certain volume of water droplets on the surfaces of three types of membrane samples. All measurements were performed under the same conditions of 0° inclination angle at room temperature, and accordingly, the measured contact angles for samples (a), (b), and (c) were confirmed to be 23.10°, 27.96°, and 34.13°, respectively. These results show that the sintering temperature has a clear effect on the change in surface hydrophilicity of the ceramic membrane. All of the membranes retained hydrophilicity, but the sample (a) sintered at the lowest temperature had higher hydrophilicity. This behavior can be further explained by the AFM roughness data, which showed that membranes sintered at lower temperatures exhibited higher surface roughness. According to the Wenzel model, increased surface roughness amplifies the intrinsic hydrophilicity of the material, leading to lower contact angles. On the other hand, the reason why the contact angle of the sintered sample (c) is the largest at the highest temperature can be expected to be that as the sintering temperature increases, the particles agglomerate and pore closure occurs, resulting in a decrease in surface roughness. The contact angle is closely related to predicting key membrane properties such as water permeability and protein adsorption behavior. In general, a lower contact angle indicates better hydrophilicity, which favors water transport and reduces nonspecific protein adsorption—both critical for hemodialysis performance. These data can be used as reference materials for applying membranes to more suitable applications. For example, sample (a) has the potential to be applied to the field of hemodialysis due to its high hydrophilicity and low protein adsorption, and a membrane with a hydrophobic surface, such as sample (c), can be used in fields such as industrial testing and wastewater treatment. Since the protein adsorption properties of the membrane are not simply due to hydrophilicity but can be affected by the roughness of the membrane, additional analysis of protein adsorption properties is essential to confirm this prediction.

Figure S3b shows the mechanical strength of the Al_2_O_3_ hollow fiber membranes. For the mechanical evaluation of hollow circular tubes, the flexural strength (σ) was calculated using the three-point bending equation for hollow cylinders:

(1) $$\sigma =\frac{32FL}{\pi ({D_{0}}^{4}-{D_{i}}^{4})}$$

In Equation (1), σ represents the flexural strength (MPa), *F* is the maximum applied force (N), *L* is the span length set to 20 mm, and *D*_0_ and *D_i_* are the outer and inner diameters of the fiber (mm), respectively. As we expected based on SEM results, the flexural strength of the membrane increased with higher sintering temperatures. This is likely due to more pronounced necking and densification between Al_2_O_3_ particles during sintering. The sample sintered at 1200 °C had an average flexural strength of 7.03 ± 0.83 MPa, which seems to result from weak particle bonding and the presence of large finger-like pores and interparticle voids, as seen in the SEM image. While this structure is beneficial for permeability, it compromises mechanical durability under external loading conditions. The mechanical strength of the sample sintered at 1300 °C was 32.19 ± 0.36 MPa, which was clearly increased compared to the previous one, as expected. This reason is thought to be the result of the aggregation of particles and the densification of the structure generated during the membrane sintering process. The mechanical strength of the sample sintered at 1400 °C was 87.74 ± 0.95 MPa, which seems to have undergone a stronger level of membrane structural change than at 1300 °C. This increase in strength secures the durability of the membrane itself, which is advantageous for stable use, but it is required to achieve an appropriate balance between the performance and strength of the target membrane because it will be disadvantageous compared to the membrane sintered at a low temperature in terms of transmittance.

Figure S3c shows the surface shape and roughness results of the Al_2_O_3_ hollow fiber membrane by sintering temperature confirmed through an atomic microscope (AFM). The upper image (Figure S3c(1–3)) is a 2D image of each sample surface, and the lower image (Figure S3c(4–6)) is a 3D image that can confirm the surface roughness. It is difficult to confirm the roughness only with the image, but the roughness (Ra) values of each sample confirmed in the analysis data values were 123 nm, 97.2 nm and 78.2 nm at sintering temperatures of 1200 °C, 1300 °C, and 1400 °C, respectively. Through this, it was confirmed that the increase in sintering temperature affects the decrease in surface roughness. The membrane sintered at 1200 °C (Figure S3c(1,4)) exhibited the highest Ra value and displayed a rougher surface with pronounced nanoparticulate texture and distinct grain boundaries. This is attributed to incomplete sintering at lower temperatures, which preserves a high surface area with well-defined topographical features. At 1300 °C (Figure S3c(2,5)), the membrane surface showed a more moderate roughness with smoother transitions between grains and a more uniform distribution of surface elevations. The sample sintered at 1400 °C (Figure S3c(3,6)) exhibited the smoothest surface, with the lowest Ra value of 78.2 nm.

### 3.2. Protein Adsorption Test Result of Ceramic Hollow Fiber Membranes

To evaluate the biocompatibility of the Al_2_O_3_ hollow fiber membranes, protein adsorption experiments were conducted using bovine serum albumin (BSA) solutions at different concentrations of 1, 5, and 10 mg/mL. The raw data for protein adsorption are shown in Table 1.

Table 2 presents the effective surface area of the Al_2_O_3_ hollow fiber membranes. Further physical specifications of the Al_2_O_3_ hollow fiber membranes used in each protein adsorption test are shown in the Supplementary Materials (Table S1).

**Figure 7:**
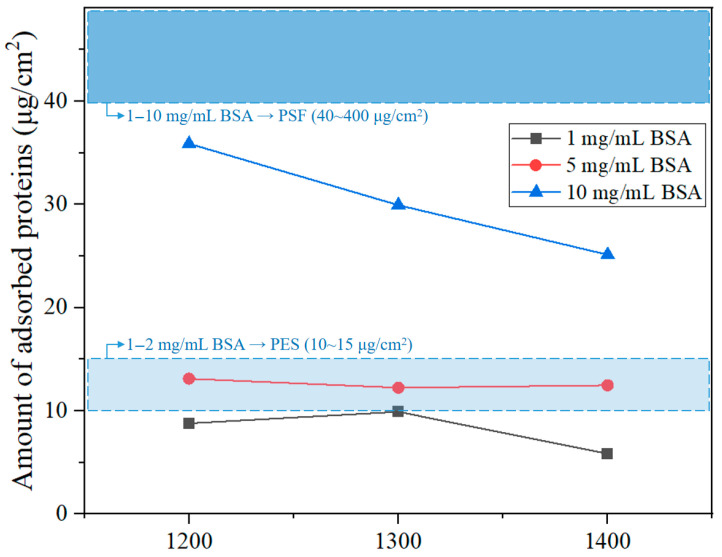
Results of protein adsorption behavior of Al_2_O_3_ hollow fiber membranes under varying BSA Concentrations: The upper blue region represents the protein adsorption of PSF membranes (approximately 40–400 μg/cm^2^) at BSA concentrations of around 1–10 mg/mL, while the lower bright-blue region indicates the protein adsorption of PES membranes (approximately 10–15 μg/cm^2^) at BSA concentrations of around 1–2 mg/mL.

For each BSA concentration, the outer diameter, inner diameter, and length of the membrane samples were precisely measured. Based on these measurements, the total surface area (cm^2^) of each membrane was calculated. Surface area values were used to normalize the amount of adsorbed protein to ensure accurate comparison of adsorption performance across samples with different geometries. The results show a typical trend in which the total amount of adsorbed protein increases with BSA concentration. However, the amount of adsorbed protein decreased with increasing sintering temperature at all concentrations. At a BSA concentration of 10 mg/mL, the membrane sintered at 1200 °C exhibited the best protein adsorption at approximately 35.88 μg/cm^2^. The membrane sintered at 1400 °C showed the lowest value of 25.12 μg/cm^2^. This suggests that higher sintering temperatures densify the surface pore structure and increase the number of closed pores, thereby hindering protein infiltration and binding. Similar trends were observed from the concentrations of 1 and 5 mg/mL. The membrane sintered at 1400 °C consistently showed the lowest adsorption. However, due to the low concentration of BSA, the amount of protein adsorbed onto the hollow fiber membranes was relatively small, making it difficult to clearly distinguish the trend observed at the 10 mg/mL condition. Figure 7 visually compares these findings, illustrating changes in adsorption levels across concentrations for each sample, along with reference ranges for commercial hollow fiber membranes made of PSF and PES. Raw data of protein adsorption test are shown in the Table S2. The Al_2_O_3_ hollow fiber membranes showed lower adsorption than PSF membranes at all concentrations and comparable to or in some cases lower levels than PES membranes under the 1 and 5 mg/mL conditions. These findings are related to the earlier contact angle and SEM results. At lower sintering temperatures, the membranes exhibited higher porosity and clearer finger-like internal structures, which could facilitate protein penetration and increase surface adsorption. In contrast, membranes sintered at higher temperatures showed denser surfaces, leading to reduced protein attachment. This tendency was consistent with AFM observations: as the sintering temperature increased, the surface became smoother, with lower Ra values. The membrane sintered at 1400 °C displayed the smoothest and most tightly packed surface, which corresponded with the lowest BSA adsorption. Interestingly, this trend differed from the prediction based on water contact angle data alone, suggesting that surface roughness had a stronger influence on protein adsorption than wettability. Overall, the Al_2_O_3_ hollow fiber membranes made in this study showed good compatibility with biological systems, and their lower protein adsorption levels outperformed those of typical commercial polymer membranes.

### 3.3. Water Flux and Permeability of Lab-Scale Hemodialyzer

**Figure 8:**
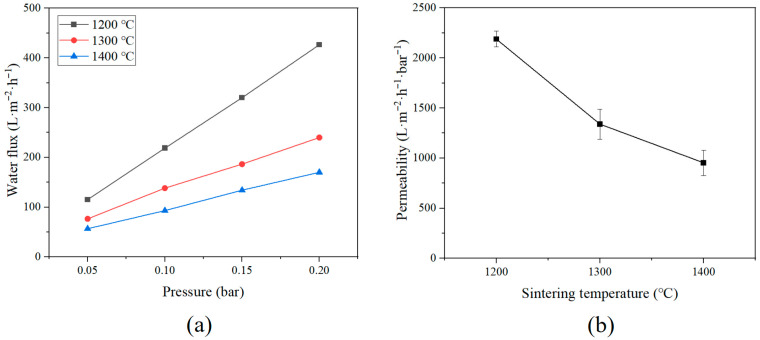
Water flux and permeability of lab-scale hemodialyzer with different sintering temperatures of Al_2_O_3_ hollow fiber membranes: (**a**) Water flux, (**b**) Permeability.

To evaluate water flux and permeability characteristics of Al_2_O_3_ hollow fiber membranes, water permeability tests were conducted using a lab-scale hemodialyzer including Al_2_O_3_ membranes made at different sintering temperatures (1200 °C, 1300 °C, and 1400 °C). Figure 8 shows two graphs: the graph on the left displays how water flow rate changes with applied pressure (0.05, 0.10, 0.15, and 0.20 bar) for each membrane, while the graph on the right compares how easily water moves through the membranes under the same pressure, depending on the sintering temperature. The water flux (*J*) of modules for each pressure was calculated using Equation (2):

(2) $$J=\frac{V}{At}$$

where *J* is the water flux (L/m^2^·h), *V* is the volume of permeated water (L), *A* is the effective membrane area (m^2^), and *t* is the measurement time (h). The volume of water passed through the membrane was calculated by Equation (3):

(3) $$V=\frac{m}{\rho }$$

where *m* is the measured weight of the permeated water (g), and ρ is the density of water (1.0 g/cm^3^ at 25 °C). The experiment was conducted at a room temperature of 25 ± 1 °C. The measurements were repeated three times for each pressure condition to obtain an average value. This approach allowed for the analysis of the water permeability characteristics of the hollow fiber membrane as a function of pressure.

All samples showed a linear increase in water flux with increasing pressure, indicating that the membranes operated within their elastic regime and that Darcy’s law was applicable under the experimental conditions [47]. However, despite identical pressure increments, a significant difference in absolute water flux was observed among the samples, depending on their sintering temperature. The water flux of the membrane sintered at 1200 °C was the highest at all pressures compared to the samples sintered at the other two temperatures. There was no significant difference at the 0.05 bar, but the difference was evident as the pressure increased, and the water flux at the highest pressure of 0.2 bar was 426.77 L·m^−2^·h^−1^, which was significantly increased than the water flux of the membrane sintered at 1300 °C and 1400 °C of 240.01 and 170.06 L·m^−2^·h^−1^. The permeability graph also showed the highest value at a low temperature, and it was confirmed that the permeability decreased as the sintering temperature increased. This decline in water transport performance correlates well with the previously discussed SEM and contact angle analyses. SEM images revealed that the membrane sintered at 1200 °C maintained a well-developed asymmetric structure with prominent finger-like voids extending from the lumen side, facilitating effective mass transport. In contrast, the membrane sintered at 1400 °C showed a much denser internal structure, where, although some finger-like features remained, their length and continuity were noticeably reduced. The contact angle measurements further support these findings. These results agree well with the results predicted through the SEM and contact angle analysis discussed previously. The thickness of the Al_2_O_3_ hollow fiber membrane, as confirmed through the SEM image, decreased as the sintering temperature increased, predicting the density of the structure, and the decrease in contact angle also supports this result well. In conclusion, the prepared membrane can predict the characteristics that change according to the sintering temperature and control them according to the necessary application.

### 3.4. Hemodialysis Performance Evaluation of Lab-Scale Hemodialyzer

**Figure 9:**
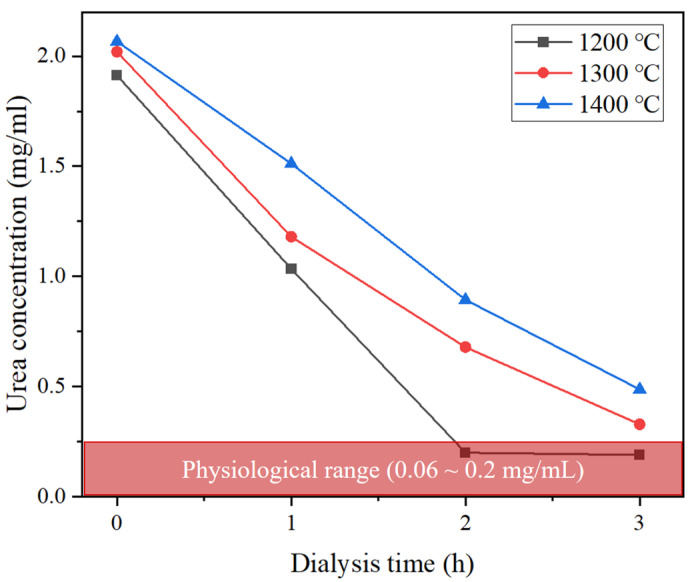
Urea removal performance of a lab-scale hemodialyzer with different sintering temperatures of Al_2_O_3_ hollow fiber membranes.

Figure 9 presents the results of urea removal performance using the fabricated Al_2_O_3_ hollow fiber membranes. The initial urea concentration was set at approximately 2.0 mg/mL. All samples exhibited a gradual decrease in urea concentration over time. In particular, the membrane sintered at 1200 °C rapidly reduced the urea concentration to below the physiological range (0.06–0.2 mg/mL) within 2 h, demonstrating the fastest removal rate. These results were obtained under recirculation conditions. This indicates that membranes sintered at lower temperatures enhance mass transfer and facilitate the diffusion of small molecules such as urea. In contrast, the membranes sintered at other temperatures did not reach the normal range even after 3 h. This is attributed to the densification of the membrane’s surface and internal structure at higher sintering temperatures, leading to reduced pore size and connectivity, which in turn limits diffusion pathways. Overall, the urea removal performance is consistent with previous findings from water flux, SEM, contact angle and AFM.

**Figure 10:**
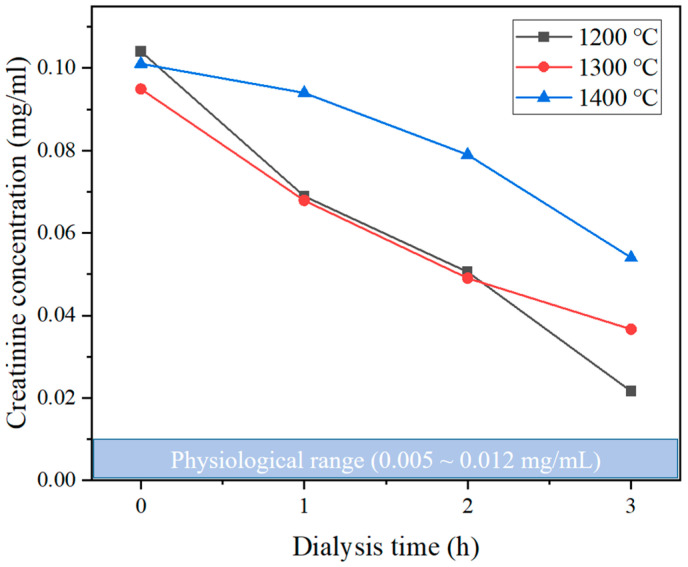
Creatinine removal performance of a lab-scale hemodialyzer with different sintering temperatures of Al_2_O_3_ hollow fiber membranes.

Figure 10 shows the creatinine removal performance of the lab-scale hemodialyzers. Unlike the urea removal experiment, all values did not reach the normal physiological range (0.005–0.012 mg/mL). The membrane sintered at 1200 °C exhibited the fastest removal rate, reducing the concentration to approximately 0.02 mg/mL after 3 h. Graph revealed a gradual decline in removal efficiency with increasing sintering temperature, with the 1400 °C sample maintaining a concentration above 0.05 mg/mL after 3 h. This outcome can be explained by the fact that the initial concentration of creatinine was significantly lower than that of urea. As the concentration fell below a certain level during the experiment, the solute concentration gradient between the blood model solution and the dialysate was no longer sufficient to sustain effective mass transfer, leading to a sharp decrease in the driving force. The relatively lower removal of creatinine can be attributed not only to the decrease in concentration gradient but also to its larger molecular weight (113 Da) compared to urea (60 Da), which results in a lower diffusivity. This difference is considered a key factor limiting creatinine transport and removal under the same conditions. Raw data of urea and creatinine removal test results are shown in the Supplementary Materials (Tables S3 and S4).

Table 3 shows the summary of hemodialysis performance metrics of lab-scale hemodialyzers. *Kt*/*V* value is used as an indicator to assess the adequacy of conventional hemodialysis. According to major international manufacturers, over a typical 3 to 4 h of hemodialysis session, the target *Kt*/*V* values are set at ≥1.4 for Fresenius Medical Care products, and between 1.2 and 1.4 for Baxter and Nipro. Therefore, a *Kt*/*V* value of 1.2 or higher is considered to indicate effective hemodialysis. In this study, the *Kt*/*V* values were calculated using Equation (4) below to compare the performance of the tested devices with that of commercial products.

(4) $$\frac{Kt}{V}$$

where *K* is the urea clearance rate (L/min), t is the time of dialysis (min), and *V* is the total body water volume (L) of the patients. To calculate the *Kt*/*V* values based on the experimental data, the urea clearance rate *K* was first determined by multiplying the blood flow rate (L/min) by the extraction ratio *E*, which is obtained using the following Equation (5).

(5) $$E=\frac{C_{in}-C_{out}}{C_{in}}$$

*E* values of 0.4597, 0.4159, and 0.2682 after one hour of dialysis of the sintered membrane at 1200 °C, 1300 °C, and 1400 °C, and *K* values calculated by multiplying the blood flow rate were 0.0184, 0.0166, and 0.0107 L/min. The *Kt*/*V* values calculated by substituting these values into Equation (4) and using the total body mass of 1.0 L are 1.10, 1.00, and 0.64, respectively. In this study, we acknowledge that the body mass volume was set to 1.0 L, which is considerably lower than that of a typical adult. This assumption was made because the membrane surface area of the lab-scale hemodialyzer used here is approximately one-tenth that of a commercial device, and accordingly, a smaller distribution volume was applied. Nevertheless, *Kt*/*V* is a normalized index that takes into account clearance, dialysis time, and membrane surface area. Thus, even though the total body water volume was set lower than in clinical reality, the calculated values remain valid for evaluating the relative removal efficiency of the device. In this context, the *Kt*/*V* values presented should be interpreted not as a direct simulation of absolute patient conditions, but rather as normalized performance indices for relative comparison of device efficiency.

The relationship between urea removal efficiency (%) and *Kt*/*V* can be used to compare the experimental performance of the developed membranes with that of commercial hemodialyzers. This value can be calculated by Equation (6) below.

(6) $$removal\;rate\;\left(\%\right)=(1-e^{\frac{-Kt}{V}})\times 100$$

This equation can be rearranged to Equation (7) to solve for *Kt*/*V*

(7) $$\frac{Kt}{V}=-\ln(1-\frac{removal\;rate\;\left(\%\right)}{100})$$

By applying the experimental data at each sintering condition, the *Kt*/*V* values were calculated as follows: 0.61 for 1200 °C, 0.53 for 1300 °C, and 0.31 for 1400 °C.

These values are lower than the *Kt*/*V* values previously obtained using direct calculation based on blood flow rate and extraction ratio (1.10, 1.00, and 0.64, respectively). This discrepancy arises from the fundamental difference between the two calculation approaches. The direct method assumes a constant removal rate and steady flow, effectively modeling urea clearance as a linear process. In contrast, the logarithmic model accounts for the fact that as dialysis progresses, the concentration gradient decreases, which reduces the driving force for diffusion. As seen in the experimental urea concentration plots, the decrease in urea concentration is not linear but gradually tapers off, supporting the validity of the logarithmic approach. Therefore, the logarithmic model reflects the real physiological kinetics of urea removal during dialysis, making it more suitable for theoretical evaluation and comparison with clinical standards.

Although these values do not meet the typical target range of commercial devices, it is important to note that such targets are generally based on dialysis treatments lasting 3 to 4 h. Therefore, the values obtained after just 60 min of treatment in this study can still be considered meaningful. In particular, considering that the membrane surface area of the lab-scale dialyzers used in this study was only about one-tenth that of commercial products, the removal efficiency per unit area is deemed to be highly competitive. Moreover, even when evaluated using a conservative calculation method, the membranes maintained a reasonable level of efficiency, supporting the practical feasibility of Al_2_O_3_ hollow fiber membranes for hemodialysis applications. However, since the contents performed in this study are limited to the laboratory-scale manufacturing process construction and performance evaluation environment, several procedures remain for actual clinical application. As a primary consideration, it is important to secure economic feasibility in the cost of mass production processes since ceramic membranes have higher raw material costs and consume more energy in the manufacturing process than polymer-based membranes. In addition, for clinical use as a new medical device, regulatory approval procedures such as safety and reliability verification must be performed. Therefore, the application of ceramic-based hemodialysis membranes as new medical devices remains a challenge to solve technical, economic, and clinical barriers in stages. Table 4. summarizes the comparative results of SEM analysis, water contact angle, AFM roughness, and mechanical strength at different sintering temperatures, highlighting the correlation between structural, surface, and mechanical properties.

## 4. Conclusions

In this study, Al_2_O_3_-based ceramic hollow fiber membranes were successfully fabricated via phase inversion and sintering processes, and their hemodialysis performance and biocompatibility were systematically evaluated through the custom-made lab-scale hemodialyzer. The membranes were sintered at 1200 °C, 1300 °C, and 1400 °C to investigate the effects of temperature on microstructure and surface properties, and how these changes influence hemodialysis performance. SEM, AFM, contact angle, and mechanical strength analyses revealed that the membrane sintered at 1200 °C maintained a well-developed finger-like asymmetric porous structure, high hydrophilicity, and a rough surface, all of which contributed to excellent water permeability and effective removal of uremic toxins. While the membrane sintered at 1400 °C showed advantages in structural stability and low protein adsorption, its mass transport performance was compromised due to pore shrinkage and reduced interconnectivity. BSA protein adsorption tests demonstrated that high-temperature sintered membranes exhibited minimal adsorption. Protein adsorption levels in all Al_2_O_3_ membranes were comparable to or even lower than those of commercial polymer membranes, indicating superior resistance to nonspecific protein fouling. These results confirm that, under the tested conditions, the ceramic membranes exhibited higher biocompatibility compared to commercial membranes. The membrane sintered at 1200 °C showed the fastest removal of both urea and creatinine and achieved a *Kt*/*V* value of 1.1 after 60 min of dialysis, approaching the level of commercial hemodialyzers. These results suggest that Al_2_O_3_ hollow fiber membranes hold significant potential as next-generation alternatives to conventional polymer-based hemodialysis membranes, although further studies on long-term stability, in vivo validation, and clinical-scale module development are required. In summary, while this work demonstrates the feasibility of ceramic membranes for hemodialysis, their clinical translation remains contingent on overcoming several key challenges. It should be noted that this study has certain limitations, including the relatively short dialysis duration compared to clinical practice and the lack of hemocompatibility tests beyond BSA adsorption. Future studies should address these aspects to more fully establish the clinical relevance of ceramic membranes. Future research must focus on reducing production costs through scalable processes and meeting the stringent safety, reliability, and regulatory requirements necessary for medical device approval.

## Figures and Tables

**Table 1:** Protein adsorption test result of Al_2_O_3_ hollow fiber membranes under varying BSA Concentrations.

BSA Concentration (mg/mL)	Sintering Temperature (°C)	Adsorbed Protein (μg/cm^2^)
1	1200	8.79
	1300	9.91
	1400	5.84
5	1200	13.12
	1300	12.24
	1400	12.48
10	1200	35.88
	1300	29.94
	1400	25.12

**Table 2:** Effective surface area of the Al_2_O_3_ hollow fiber membranes for protein adsorption test.

BSA Concentration (mg/mL)	Sintering Temperature (°C)	Surface Area (cm^2^)
1	1200	2.42
	1300	2.53
	1400	2.37
5	1200	2.46
	1300	2.65
	1400	2.41
10	1200	2.74
	1300	2.64
	1400	2.32

**Table 3:** Summary of urea removal efficiency and dialysis performance metrics (*E*, *K*, *Kt*/*V*) for lab-scale hemodialyzer including Al_2_O_3_ hollow fiber membranes sintered at different temperatures.

Sintering Temperature (°C)	1200	1300	1400
Removal rate (%) (h = 1)	45.96	41.58	26.82
Initial concentration (mg/dL)	191.321	202.014	206.665
Extraction ratio (*E*)	0.4596	0.4155	0.2681
*K* (L/min)	0.0184	0.0166	0.0107
*Kt*/*V* (direct) (t = 60)	1.10	1.00	0.64
*Kt*/*V* (log model) (t = 60)	0.61	0.53	0.31

**Table 4:** Summary of morphological, surface, and mechanical properties of Al_2_O_3_ hollow fiber membranes sintered at different temperatures.

Sintering Temperature (°C)	1200	1300	1400
SEM observation	Finger-like pores, loose bonding	Reduced pores, denser structure	Dense, compact structure
Contact angle (°)	23.10	27.96	34.13
AFM Ra (nm)	123	97.2	78.2
Flexural strength (MPa)	7.03 ± 0.83	32.19 ± 0.36	87.74 ± 0.95

## Data Availability

The original contributions presented in this study are included in the article. Further inquiries can be directed to the corresponding author.

## Supplementary Materials

The following supporting information can be downloaded at: https://www.mdpi.com/article/10.3390/membranes15090251/s1. Table S1: Physical specifications of the Al_2_O_3_ hollow fiber membranes for the BSA protein adsorption test. Table S2: Raw data of protein adsorption test of Al_2_O_3_ hollow fiber membranes under varying BSA Concentrations. Table S3: Raw data of the urea removal test result of the lab-scale hemodialyzer. Table S4: Raw data of creatinine removal test results of lab-scale hemodialyzer. Figure S1: Preparation of ceramic hollow fiber membranes. (a) schematic illustration of the fabrication process, (b) photographic image of the spinning setup and coagulation bath, (c) sintering profiles for ceramic hollow fiber membranes. Figure S2: BSA calibration for protein adsorption test. (a) photographic image of the BSA calibration process, (b) BSA calibration curve in the concentration range of 0–1.0 mg/mL. Figure S3 (a) Static water contact angle measurements of Al_2_O_3_ hollow fiber membranes sintered at different temperatures, (b) The mechanical strength of the Al_2_O_3_ hollow fiber membranes, (c) AFM images of Al_2_O_3_ hollow fiber membranes sintered at different temperatures: (1–3) 2D surface topography and (4–6) corresponding 3D surface profiles of membranes sintered at (1,4) 1200 °C, (2,5) 1300 °C, and (3,6) 1400 °C.
