# Supplementary material for: Fabrication and Evaluation of Ceramic-Based Hollow Fiber Membrane Modules for Hemodialysis Applications
Source: Membranes (Basel). 2025 Aug 26;15(9):251. doi: 10.3390/membranes15090251 (PMC12471399; doi:10.3390/membranes15090251)
Supplement: Supplementary file 1 [file membranes-15-00251-s001.zip › membranes-3822081-supplementary.pdf]

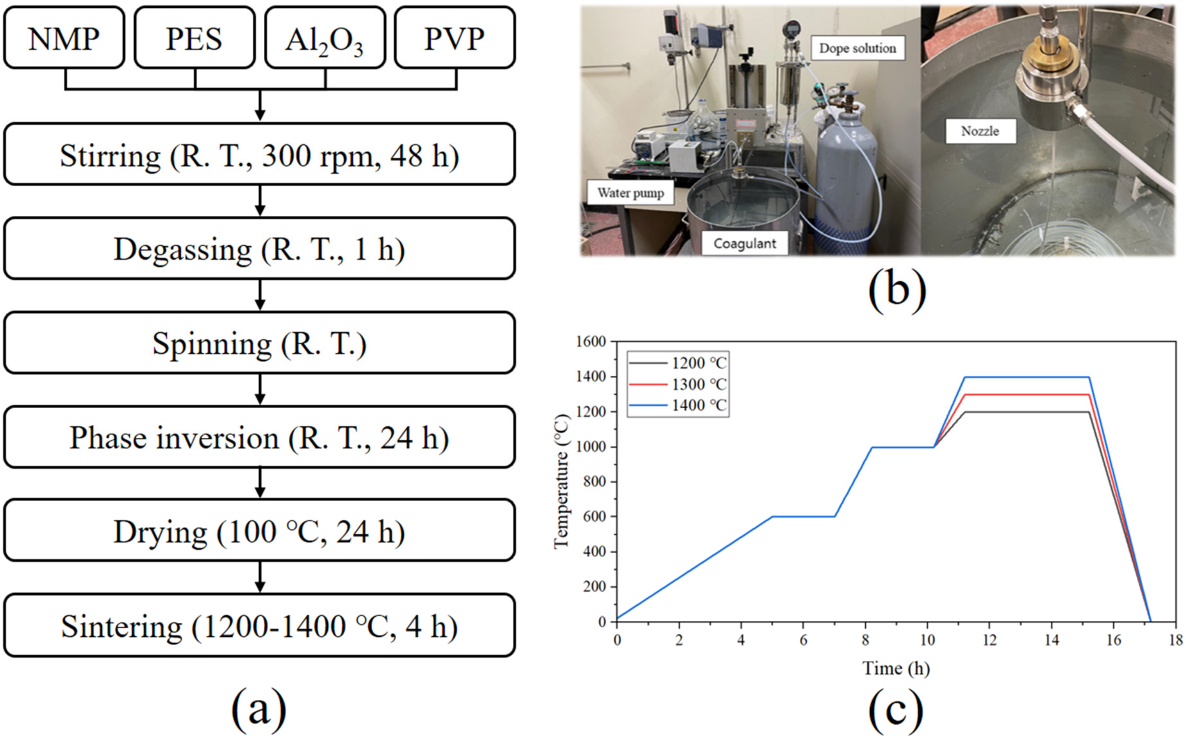

<Figure S1> Preparation of ceramic hollow fiber membranes. (a) schematic illustration of the fabrication process, (b) photographic image of the spinning setup and coagulation bath, (c) sintering profiles for ceramic hollow fiber membranes.

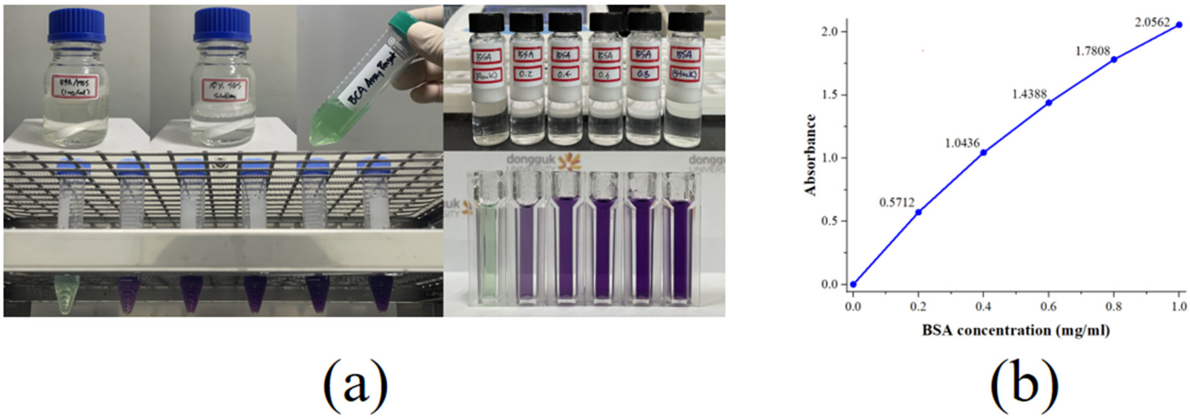

<Figure S2> BSA calibration for protein adsorption test. (a) photographic image of the BSA calibration process, (b) BSA calibration curve in concentration range of 0-1.0 mg/ml

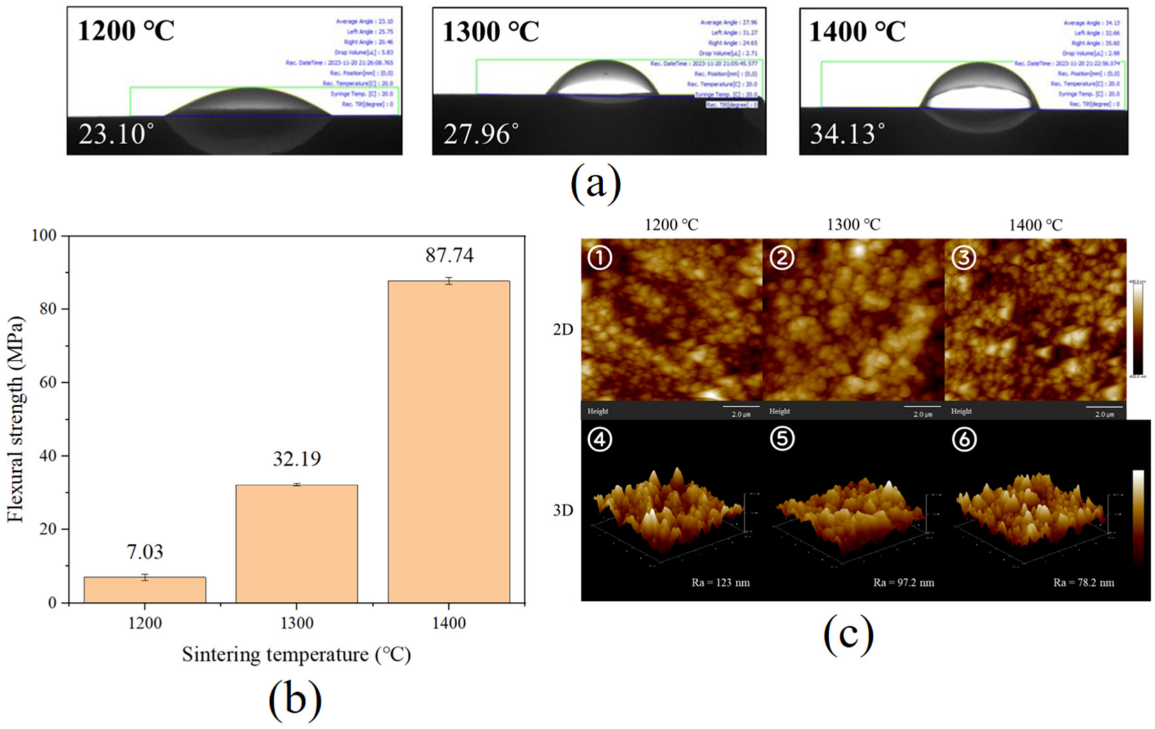

<Figure S3> (a) Static water contact angle measurements of Al_2O_3 hollow fiber membranes sintered at different temperatures, (b) The mechanical strength of the Al_2O_3 hollow fiber membranes, (c) AFM images of Al_2O_3 hollow fiber membranes sintered at different temperatures: (1-3) 2D surface topography and (4-6) corresponding 3D surface profiles of membranes sintered at (1, 4) 1200 °C, (2, 5) 1300 °C, and (3, 6) 1400 °C.

<Table S1> Physical specifications of the Al_2O_3 hollow fiber membranes for BSA protein adsorption test

BSA (mg/ml)	Sintering Temperature (℃)	Outer diameter (mm)	Inner diameter (mm)	Length (mm)	Surface area	
					(mm^2)	(cm^2)
1	1200	2.26	1.48	20.2	241.92	2.42
	1300	2.39	1.54	20.1	253.41	2.53
	1400	2.18	1.48	20.3	237.43	2.37
5	1200	2.29	1.56	20.0	246.31	2.46
	1300	2.41	1.70	20.2	265.40	2.65
	1400	2.19	1.54	20.2	240.51	2.41
10	1200	2.43	1.65	21.0	274.17	2.74
	1300	2.45	1.69	19.9	263.76	2.64
	1400	2.19	1.54	19.5	232.31	2.32

<Table S2> Raw data of protein adsorption test of Al_2O_3 hollow fiber membranes under varying BSA concentrations.

BSA	Sintering	Absorbance	BSA concentration	adsorbed

(mg/ml)	Temperature (℃)	(562 nm)	(mg/ml)	(μg/ml)	protein (μg/cm^2)
1	1200	0.0650	0.0213	21.3	8.79
	1300	0.0763	0.0251	25.1	9.91
	1400	0.0428	0.0139	13.9	5.84
5	1200	0.0977	0.0322	32.2	13.12
	1300	0.0982	0.0324	32.4	12.24
	1400	0.0912	0.0300	30.0	12.48
10	1200	0.289	0.0983	98.3	35.88
	1300	0.234	0.0790	79.0	29.94
	1400	0.174	0.0582	58.2	25.12

<Table S3> Raw data of urea removal test result of lab-scale hemodialyzer

Sintering Temperature (℃)	Urea concentration (mg/ml) by time (h)			
	0 h	1 h	2 h	3 h
1200	1.913	1.034	0.199	0.189
1300	2.020	1.180	0.679	0.329
1400	2.067	1.512	0.894	0.487

<Table S4> Raw data of creatinine removal test result of lab-scale hemodialyzer

Sintering Temperature (℃)	Urea concentration (mg/ml) by time (h)			
	0 h	1 h	2 h	3 h
1200	0.104	0.069	0.051	0.022
1300	0.095	0.068	0.049	0.037
1400	0.101	0.093	0.079	0.054
